# Enhancement of Electrochromic Properties of Polyaniline Induced by Copper Ions

**DOI:** 10.1186/s11671-022-03689-1

**Published:** 2022-05-12

**Authors:** Ting Qin, Lianwen Deng, Pin Zhang, Min Tang, Chen Li, Haipeng Xie, Shengxiang Huang, Xiaohui Gao

**Affiliations:** 1 School of Physics and Electronics, Central South University, Changsha, 410083 China; 2 National Key Laboratory on Electromagnetic Environmental Effects and Electro-Optical Engineering, Army Engineering University of PLA, Nanjing, 210007 China

**Keywords:** Polyaniline, Electrochromic device, Infrared emissivity, Polarons and bipolarons

## Abstract

Driven by the urgent need for adaptive infrared (IR) electrochromic devices, the improvement in electrochromic performance based on polyaniline (PANI) conducting polymers has become an outstanding challenge. In recent years, the acid doping strategy has been proven to increase the IR modulation ability of PANI, in particular for the Bronsted acid doping. Herein, the effects of copper ions, a Lewis acid, on the structure and electrochromic properties of polyaniline were investigated. Compared to pure polyaniline, the Cu-doped PANI porous films show better IR modulation ability. With the increasing concentration of copper ions, the Cu-doped PANI porous films exhibit a trend in volcanic patterns for the emittance variation (∆*ε*), depending on the number of polarons and bipolarons. The optimal IR emissivity (*ε*) modulation obtained on Cu-doped PANI films shows the ∆*ε* modulation of 0.35 and 0.3 in the wavelength range of 8–14 µm and 2.5–25 µm, superior to previously reported pure sulfuric acid-doped PANI. Furthermore, a flexible IR electrochromic device was fabricated with the present Cu-doped PANI porous films. The modulation of the emittance variation varied between 0.513 and 0.834 (∆*ε* = 0.32 in ranges of wavelength 8–12 µm), suggesting the great potential for applications in military camouflage and intelligent IR thermal management. We believe that the results in this work will provide a novel perspective and avenue for improving the IR modulation ability of electrochromic devices based on polyaniline conducting polymers.

**Supplementary Information:**

The online version contains supplementary material available at 10.1186/s11671-022-03689-1.

## Introduction

Electrochromism is a phenomenon in which the optical properties of materials, such as absorbance and transmittance, can be tuned reversibly by applying a small potential. Electrochromic (EC) materials and devices that respond in the mid-to-far IR region are very important, because of their wide applications in smart windows, infrared camouflage, and thermal IR optical switches [1–6]. The commonly reported materials mainly include transition metal oxides (WO_3_ [7–9]) and conducting polymers (CPs) (polythiophene [10], polypyrrole [11], and polyaniline [12, 13]). Among these, polyaniline (PANI) has shown good spectral modulation characteristics due to its tunable structure and reversible redox properties. Meanwhile, the doping strategy has been proved to effectively improve the IR electrochromism of PANI systems. For example, P. Chandrasekhar et al*.* [14] first put forward the poly(anethosulfonate)-doped PANI films on the gold substrates as a functional layer of the electrochromic device. The excellent IR emissivity (*ε*) of the polymeric matrix-doped PANI varied from 0.32 to 0.79 in ranges of wavelength 0–40 μm. However, the used macromolecular polymer dopant is very expensive. Following this work, Li et al*.* [15] investigated the performances of the electrochromic devices that were prepared by using H_2_SO_4_-doped PANI films as the electrodes. The devices showed modulation of the emittance variation (∆*ε*) of 0.24 in ranges of wavelength 8–12 μm. Topart et al*.* prepared a non-flexible IR electrochromic device based on the camphor sulfonic acid (CSA)-doped PANI film deposited by spin coating, which showed the dynamic emittance variation from 0.35 to 0.8 at 12 μm [16]. Lu et al*.* demonstrated that high ∆*ε* (0.559 and 0.39 in ranges of wavelength 3–5 μm and 8–14 μm, respectively) of CSA-doped PANI porous films is more easily obtained by the substrates with small pore size [17]. In addition, Y. Li’ s [12, 13, 18–20] group applied different acids as the dopants to prepare the PANI films and assembled them into the device, achieving the optimal Δ*ε* of 0.43 and 0.4 at the wavelength range of 8–14 μm and 2.5–25 μm, respectively. Despite these achievements, the study on IR electrochromic mechanism of PANI is still restrictive and the new strategies and electrochromic mechanism analysis is important.

As reported, polarons and bipolarons are the main carriers in the conductive polymer chain, and the optical properties of conductive polymers are determined by the new carrier state generated in the energy gap. The state of polarons and bipolarons largely affects the variable IR emissivity of PANI films. In Chandrasekhar’s work [14], the different IR transmittances of the overlying PANI layer under different oxidation states affected the reflection from the underlying Au layer. By analyzing different reflection and *ε* values of the PANI Au/porous substrate films, it demonstrated that the IR spectral regions of PANI are mainly contributed by bipolaron states of PANI [21]. Zhang et al*.* [6] also reported that the occurrence and disappearance of polarons and bipolarons delocalizing on the PANI chains were the key parameters affecting the variable IR emissivity of PANI films, which was illustrated by analyzing the electrochemical oxidation/reduction process and structural evolution of PANI. Meanwhile, the part of the PANI film without participating in the redox reaction (called inactive PANI) was revealed to result in higher actual emissivities. As is well known, metal ions, as a Lewis acid, can coordinate with nitrogen atoms to modulate carriers transfer and contribute to catalyzing the reaction for inactive PANI. Therefore, it is believed that numerous polarons and bipolarons delocalized on the PANI chains will be presented by doping of copper ions.

In this paper, the Cu-doped PANI porous films were prepared by electropolymerization on an Au/PES porous substrate. The optimal ∆*ε* of Cu-doped PANI films achieves 0.35 and 0.3 in ranges of wavelength 8–14 µm and 2.5–25 µm, respectively. Compared to the traditional acid-doped PANI films, the stronger IR regulation ability from the present Cu-doped PANI porous film is attributed to the more polarons and bipolarons delocalized on the PANI chains. The switching times of the Cu-doped PANI porous films for the coloration and bleaching are 1.5 s and 0.7 s, respectively. Moreover, an IR electrochromic device constructed with Cu-doped PANI Au/porous substrate films exhibits a higher IR modulation ability (∆*ε* = 0.32 in the wavelength range of 8 to 12 µm). The IR electrochromic device developed in this work has a potential application in IR stealth and spacecraft thermal control.

## Materials and Methods

### Materials

Aniline (99.5%, AR) was purchased from Aladdin Biochemical Technology Co. Ltd. and distilled under reduced pressure before using it. Microporous substrates of polyethersulfone (PES) with a pore size of 1 μm and polyethylene (PE) with 5-mm-thick films were obtained from Aladdin Biochemical Technology Co. Ltd. A 200-nm-thick Au layer was deposited on the PES porous substrate by thermal evaporation. Copper sulfate (CuSO_4_, 99%, AR) and polyvinyl alcohol (PVA, average MW 105,000) were received from Alfa Aesar Company. Ultrapure water was prepared with an AquaPro system (18.2 MΩ.cm).

### Preparations of Cu-Doped PANI Porous Films

The synthesis of Cu-doped PANI films was completed through the electropolymerization on the Au/PES microporous membrane (1 × 1 cm^2^) in an aqueous solution of 1 M H_2_SO_4_, 0.5 M aniline monomer, and different content doping of CuSO_4_ (0 M, 0.005 M, 0.01 M, 0.02 M, 0.03 M, respectively) by a chronoamperometry method at the potential of 0.65 V and a polymerization charge of 0.37 C/cm^2^. An Ag/AgCl and Au/PES microporous membrane (2 × 2 cm^2^) was used as the reference electrode and counter electrode, respectively. After the electropolymerization, the prepared films were washed with ultra-pure water and then air-dried at the temperature of 40 °C. The final product was marked PANI-0, PANI-0.5, PANI-1, PANI-2, and PANI-3 depending on the copper ion concentration, respectively.

### Fabrication of Electrochromic Device

**Scheme 1:**
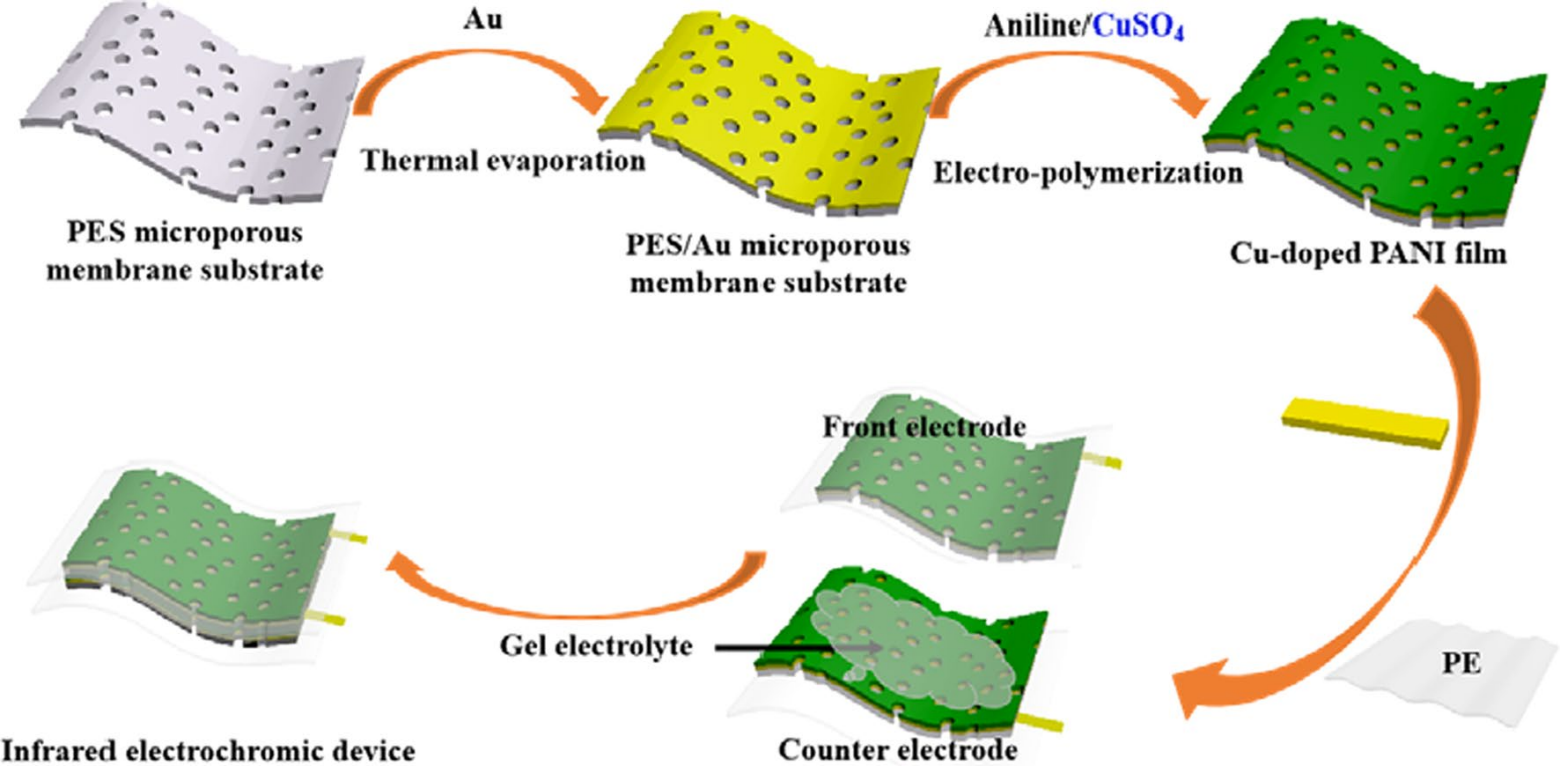
Schematic illustration of the fabrication process for the Cu-doped PANI porous films and electrochromic device

A device with a “sandwich” structure was assembled using the Cu-doped PANI porous films on the Au/PES substrate as both the front and back electrodes. Scheme 1 shows the route followed for the assembly of the device. Firstly, a 5-mm-thick IR-transparent encapsulant layer of PE was heat-bonded directly to the surface of the front electrode, protecting the PANI functional layer from the external environment. Subsequently, the back of the front electrode and the surface of the counter electrode were coated with a gel electrolyte and allowed to dry over 72 h. (The electrolyte comprised PVA, sulfuric acid, and ultrapure water in 12%/6%/82% weight ratio. The electrolyte mixture was heated to 80 °C until the PVA was dissolved. The electrolyte was reheated before use.) Finally, the working electrode and the counter electrode with the gel electrolyte were assembled in a two-electrode configuration by hot-pressing at 150 °C.

### Characterizations

The scanning electron microscopy (SEM) measurements were operated on a Hitachi S4800 with a working accelerating voltage of 10 kV. The surface morphology of the device was examined with an atomic force microscope (AFM 5500, Agilent Technologies). X-ray diffraction (XRD) measurements were conducted on a Rigaku D Max 2500 Powder Diffractometer with Cu-Kα radiations (*λ* = 0.15406 nm). Raman spectra were recorded using an inVia Qontor (Renishaw, UK) system at the wavelength of 532 nm. X-ray photoelectron spectroscopy (XPS) measurements were performed on an AVG Thermo ESCALAB 250 spectrometer (VG scientific) system using monochromatic Al-Kα radiation (*hν* = 1486.6 eV) from an X-ray source operating at 400 µm spot size, 25 W power, and 15 kV acceleration voltage. The high-resolution XPS spectra were collected with the hemispherical analyzer at the pass energy of 30 eV, the energy step size of 0.05 eV, and the photoelectron takeoff angle of 45° concerning to the surface. The Shirley background subtraction and the peak fitting with Gaussian–Lorentzian-shaped profiles were performed for the high-resolution XPS spectra analysis. The XPS data were calibrated to the C1s peak and analyzed using CasaXPS software.

### Measurements

The electrochemical measurements of Cu-doped PANI porous film were performed in a three-compartment system with 0.5 M H_2_SO_4_ aqueous solutions as the electrolyte. An Ag/ AgCl electrode and a graphite rod were utilized as the reference electrode and counter electrode, respectively. For the device, the electrochemical tests were conducted in a system with a two-electrode configuration. Cyclic voltammetric (CV) and chronoamperometric (CA) experiments were carried out on the electrochemical workstation (CHI760E, Shanghai Chenhua Instruments, China).

IR reflectance and transmittance spectra of the Cu-doped PANI films at different voltages were carried out by Nicolet IS50 Fourier IR spectroscopy (America). Owing to the opaque nature of the PANI porous film, the value of IR emissivity of the Cu-doped PANI films and devices can be calculated by weighting [1 − *R*(*λ*)] (namely spectral emittance) with the black body spectrum for a particular wavelength and integrating this in a spectral range of 2.5 to 25 µm according to the following two equations [22, 23]:

1 $$B(\lambda ) = \frac{{2\pi hc^{2} }}{{\lambda^{5} }}\frac{1}{{e^{{(\frac{hc}{{\lambda kT}}) - 1}} }}$$

2 $$\varepsilon = \frac{{\int\limits_{\lambda \min }^{\lambda \max } {(1 - R(\lambda )) \cdot B(\lambda )d_{\lambda } } }}{{\int\limits_{\lambda \min }^{\lambda \max } {B(\lambda )d_{\lambda } } }}$$

where *h* is the Planck constant (6.6261 × 10^−34^ J s), *c* is the speed of light (2.998 × 10^8^ m s^−1^), *λ* is the wavelength, *T* is the temperature (K), *B*(*λ*) is the spectral radiance of the black body at *λ,* and *R(λ)* is the reflectivity at *λ*.

## Results and Discussion

Scheme 1 displays the synthetic procedures of Cu-doped PANI porous films and corresponding devices. The Cu-doped PANI porous films were obtained by in situ electrochemical depositions of aniline molecules in the H_2_SO_4_ aqueous solution. The Au porous substrate layer has two functions: One is to provide a conductive framework and external electrode for the electrode of IR electrochromic device; second, the gold metal has a high reflectivity in the IR range, which can effectively reflect the IR energy through the working electrode of PANI films. When polymerized in acid dopant, the PANI begins to aggregate on the surface of the Au/PES porous membrane. Meanwhile, the color of the membrane surface changes from golden to yellowish-green and to dark green as the polymerization charge increases.

**Fig. 1:**
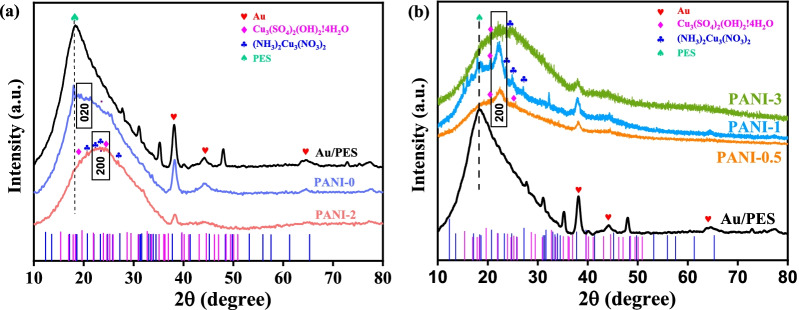
**a** The XRD image of Au/PES substrate, PANI-0, and PANI-2 porous films. **b** PANI-0.5, PANI-1, and PANI-3 porous films

To reveal the composition information, the XRD patterns are collected. As shown in Fig. 1, all the samples present a wide peak centered at 18.5°, which could be from the amorphous structure of the PES substrate [24]. According to the previous reports, the different diffraction peak usually relates to the mono-distributions of the periodicity between the polymer backbone chains [25]. For the pure PANI, a broad peak at 2θ = 20° is assigned to the periodicity parallel to the polymer chains of PANI (020). The Cu-doped PANI film shows a diffraction peak at 2θ = 25°, indicating the periodicity perpendicular to the polymer backbone chain (200) [26]. With the introduction of copper ions, the PANI signal peaks from Cu-doped PANI porous films become stronger, suggesting that Cu ions stimulate an increase in crystalline fraction and formation of Cu/PANI co-crystals. Furthermore, it is found that two signal peaks from Cu_3_(SO_4_)_2_(OH)_2_·4H_2_O and (NH_3_)_2_Cu(NO_3_)_2_ exist in the prepared Cu-doped PANI porous films, as marked by the diamonds and clubs (PDF# Cu_3_(SO_4_)_2_(OH)_2_·4H_2_O and PDF# (NH_3_)_2_Cu(NO_3_)_2_). It indicates the possible existence of Cu–N coordination bonds in Cu-doped PANI porous films, meantime the simultaneous doping of sulfuric and nitric acid ions. The signal peaks of (NH_3_)_2_Cu(NO_3_)_2_ from PANI-1 and PANI-2 porous films are the sharpest among the prepared PANI porous films, which may have a great impact on the subsequent electrochromic properties.

**Fig. 2:**
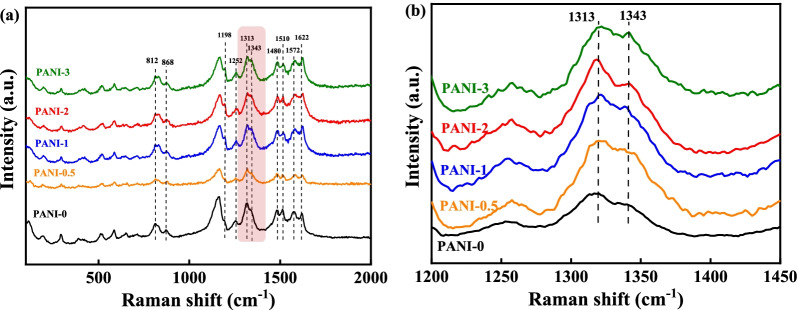
**a** Raman spectra of the PANI porous films at different copper concentrations. **b** Raman spectra of the partial enlargement of the red area of Fig. 1a

To analyze the chemical bonds in the PANI films, the Raman spectra were measured in the range of 100–2000 cm^−1^. In Fig. 2, all the Cu-doped PANI porous films present the signals at the same positions. Specifically, the characteristic peaks observed at 1622 and 1572 cm^−1^ are assigned to the C–C stretching vibration of the benzenoid ring and C=C stretching vibration of the quinoid ring, respectively. The peaks at 1480, 1313, and 1252 cm^−1^ in the spectra are attributed to the C=N stretching vibration of the quinoid ring and the C–N stretching vibrations of the benzenoid ring and quinoid ring, respectively. The C–N^·**+**^ stretching vibration of more delocalized polaronic structures is represented by the peak at 1343 cm^−1^ (Fig. 2b), and the benzenoid ring deformation in polarons is suggested by the signal at 868 cm^−1^ [27]. Compared with the Raman spectrum of the PANI-0 porous film, the Cu-doped PANI porous films show a stronger and sharper absorption peak at 1343 cm^−1^. As expected, this reveals that a large number of polarons and bipolarons delocalized on the PANI chains with the introduction of copper ions. The peaks at 1198 cm^−1^ and 812 cm^−1^ represent the C-H in-plane bending vibration and out-plane bending vibration in the quinoid ring [28]. Note that the intensity of the peak at 1198 cm^−1^ increases with the introduction of copper ions, suggesting the increased oxidation degree of Cu-doped PANI porous films.

**Fig. 3:**
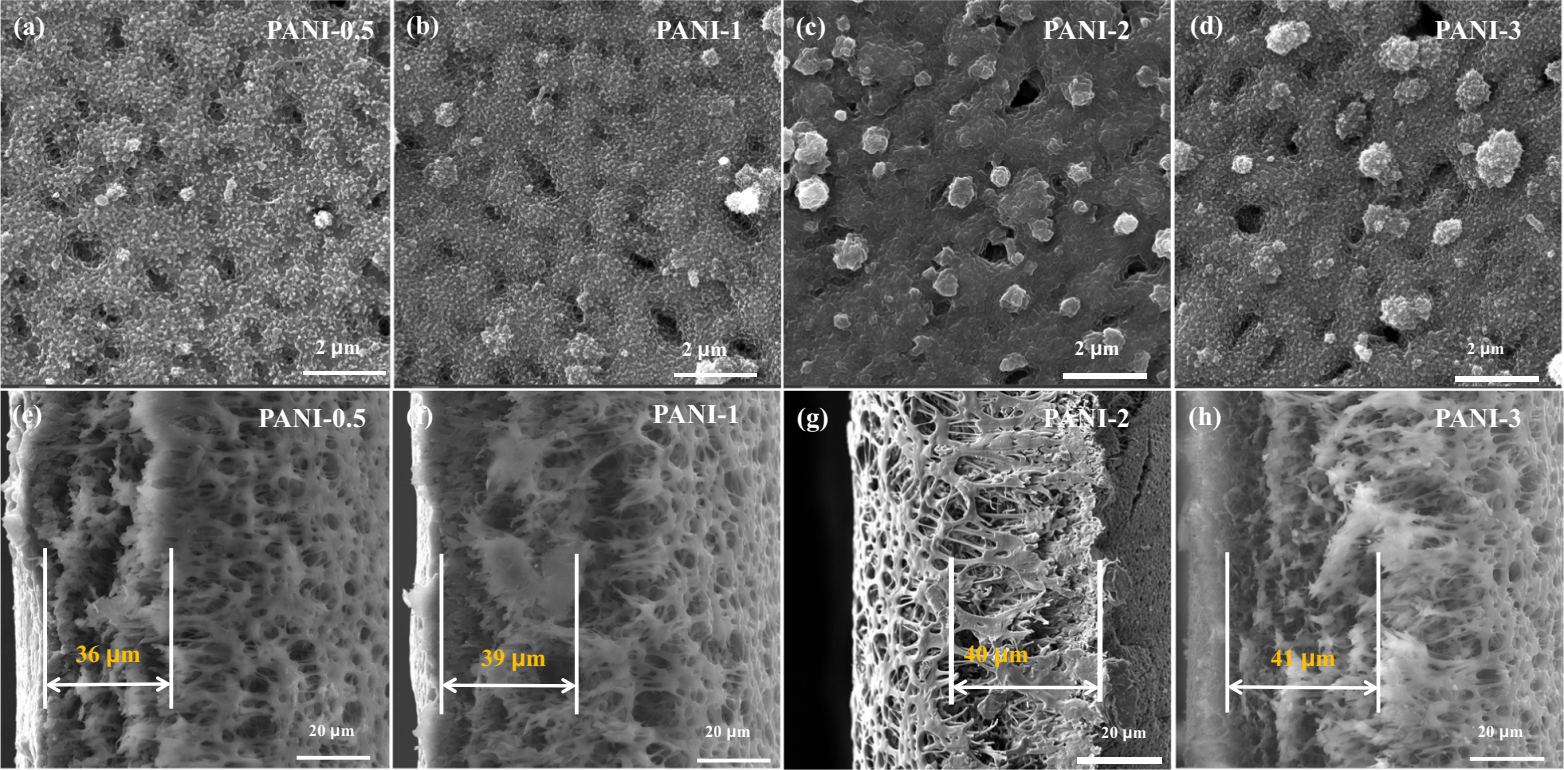
SEM images of surface on **a** PANI-0.5, **b** PANI-1, **c** PANI-2, **d** PANI-3 porous films, and cross sections of **e** PANI-0.5, **f** PANI-1, **g** PANI-2, **h** PANI-3 porous films

The geometric structure of the material is critical to optical response performance since the ion transfer between the electrode material and electrolyte plays a decisive role during the electrochemical redox process. Herein, the morphologies of PANI porous films are investigated by SEM images. As shown in Additional file 1: Fig. S2, similar to that of the PES and Au/PES membranes with a linear porous network structure, pure PANI film presents a fibrous structure, which constructs a reticular membrane. The porous structure benefits to electrolyte infiltration and ion transmission. The surface morphology of the Cu-doped PANI porous films is exhibited in Fig. 3a–d. Figure 3e–h shows the cross sections of the prepared Cu-doped PANI films. Obviously, all the PANI layers with a thickness of 40 µm are tightly attached to the Au/PES porous membrane, and uniform voids can be found in the porous structures. Additional file 1: Figure S1 shows the polymerization current as a function of time for prepared PANI porous films (i–t curves). During the entire electrodeposition process, the aggregation process of PANI on the porous films can be described in three stages. At the beginning of the polymerization reaction, it is mainly performed through linear polymerization. PANI is gradually filled on the walls of the porous membrane, constructing a reticular membrane similar to the morphology Au/PES membrane. Subsequently, with increasing polymerization time, the polymerization mode was changed from linear polymerization to radial polymerization. The PANI film grows perpendicular to the polymer backbone chain, and the cavities of the porous films are gradually filled by PANI particles. Finally, large particles and rodlike PANI appear on the surface of the films and are accumulated. Moreover, with the addition of copper concentration, the surface morphologies of Cu-doped PANI porous films become more compact and rougher (Fig. 3), indicating that the cavities of the porous films were gradually filled by PANI nanoparticles and crystal complexes visible on the XRD patterns.

**Fig. 4:**
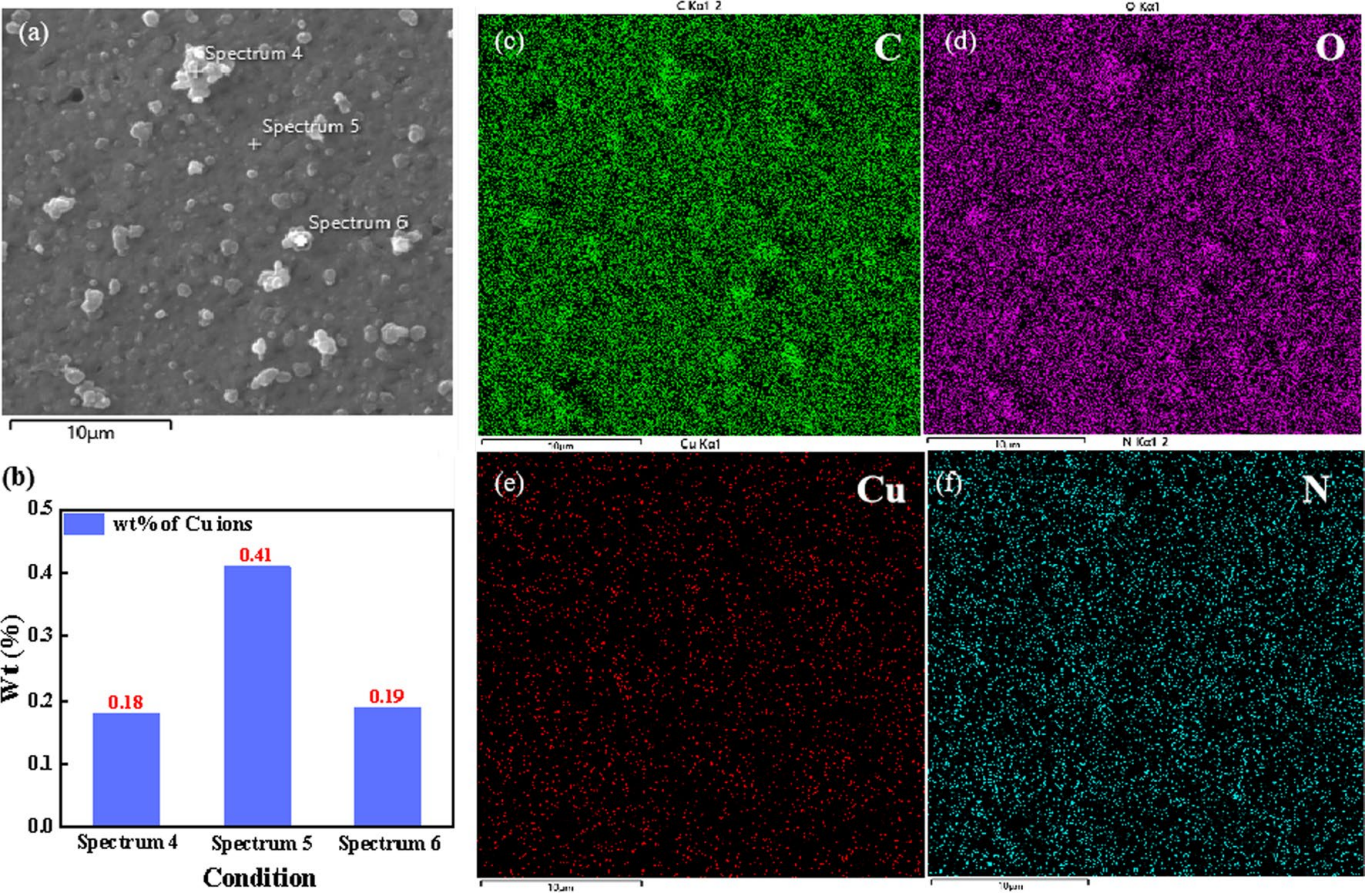
**a** SEM images of PANI-2 porous film, **b** copper ions content of the above area, EDS elemental mapping images of the: **c** C element, **d** O element, **e** Cu element, **f** N element

To present the surface roughness of PANI porous films, the AFM was used to investigate the topography of Cu-doped PANI films. Additional file 1: Figure S3 depicts 2D and 3D images of different copper contents for Cu-doped PANI film in area 5 × 5 µm^2^. The ridges and the valleys on the surface of porous films can be observed from the layers of prepared films. The roughness *Ra* values are calculated to be 69.8 nm, 85.5 nm, 115.7 nm, and 135.4 nm for PANI-0.5, PANI-1, PANI-2, and PANI-3 porous films, respectively. The energy-dispersive spectrometer (EDS) mapping images of Cu-doped PANI film surface are shown in Fig. 4a, c–f. The results show that the elements of carbon, oxygen, copper, and nitrogen are uniform distribution on the surface of PANI-2 porous films. Compared to that in spectrum 4/6, the higher copper content in spectrum 5 indicates that the copper element is distributed uniformly in the prepared films, rather than the local larger nanoparticles, as shown in Fig. 4b.

**Fig. 5:**
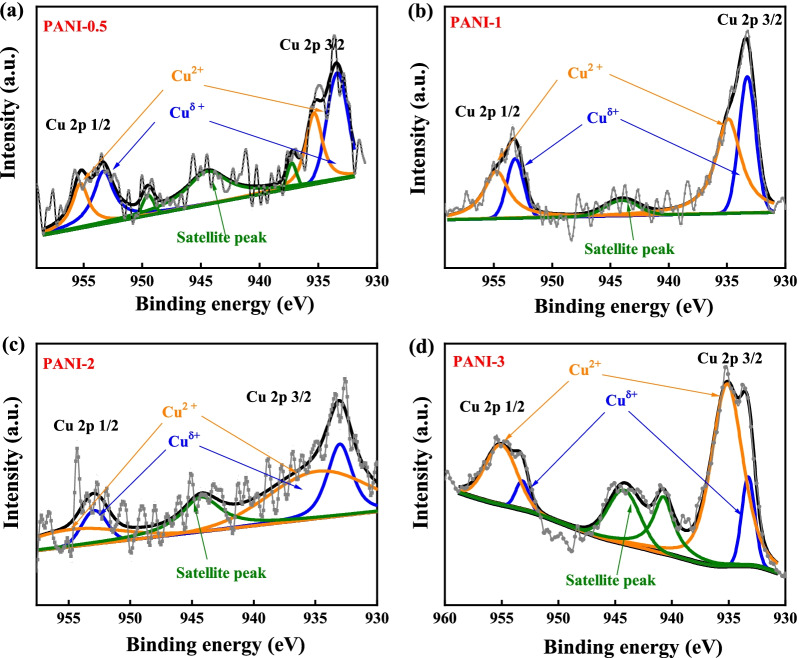
XPS spectra of Cu 2p spectrum for **a** PANI-0.5, **b** PANI-1, **c** PANI-2, and **d** PANI-3 porous films

To reveal the elements’ electronic state, XPS analyses were conducted. As shown in Additional file 1: Fig. S4, all the Cu-doped PANI films show the presence of carbon, nitrogen, oxygen, sulfur, and copper element. As a comparison, the copper signals are missing in PANI-0 films. To analyze the chemical valence state of the copper elements and disclose their role on polarons, the high-resolution Cu 2p spectra are exhibited in Fig. 5. Benefitting from the sensitivity of X-ray photoelectron energy spectra toward the element coordination environment, the copper signals from all the Cu-doped PANI films can be fitted into two components with the presence of two satellite peaks. The binding energies near 934.98 and 955.14 eV are originated from the characteristic peaks of Cu 2p_3/2_ and Cu 2p_1/2_ from Cu (II) species, while the two peaks located at 932.3 and 952.05 eV correspond to the Cu 2p_3/2_ and Cu 2p_1/2_ from Cu(I) species, respectively [29–31]. Therefore, the binding energies near 933 and 953 eV are originated from the characteristic peaks of Cu 2p_3/2_ and Cu 2p_1/2_ from Cu(*δ*) species. The Cu^δ+^ (+ 1 < δ <  + 2) with a lower oxidation state could be from Cu–N coordination bonds, and the Cu (II) species come from the metal salt. These are inconsistent with those from the above XRD. As shown in Table 1, with the increase in Cu content, the Cu^δ+^ species of prepared PANI films shift to lower binding energy and then to higher energy. Such behavior suggests that the introduction of copper ions not only facilitates protonation of quinonoid imine, but also forms Cu–N coordination compounds in polymer films. As reported, Cu–N coordination bonds were also catalyzed REDOX reactions [32]. Therefore, the Cu–N coordination bonds formed in PANI-2 can obtain the best electrochromic performance.

**Table 1:** Binding energies of Cu 2p spectrum for Cu-doped PANI porous films

	Cu 2p_3/2_		Cu 2p_1/2_	
	Cu^2+^	Cu^δ+^	Cu^2+^	Cu^δ+^
PANI-0.5	935.36 eV	933.39 eV	955.16 eV	953.21 eV
PANI-1	934.89 eV	933.27 eV	954.89 eV	953.17 eV
PANI-2	934.82 eV	**933.01 eV**	954.72 eV	**952.81 eV**
PANI-3	935.13 eV	933.27 eV	955.03 eV	953.16 eV

**Fig. 6:**
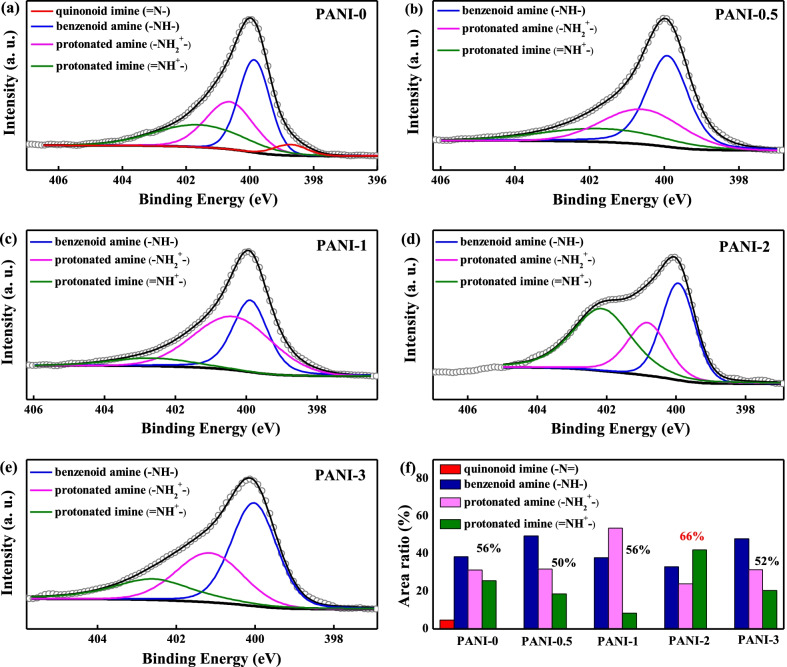
XPS spectra of N 1 s spectrum for **a** PANI-0, **b** PANI-0.5, **c** PANI-1, **d** PANI-2, **e** PANI-3 porous films, and **f** the ratio of nitrogen species to total nitrogen content for N 1s spectrum

As the coordinate atoms, the fine analysis of the N 1 s core-level spectra was performed. As shown in Fig. 6a–e, all the spectra from PANI films can be fitted into three major components located at 398.5, 399.5, 401.1, and 402.2 eV, which are attributed to the quinonoid imine (=N–), benzenoid amine (-NH-), protonated amine (-NH_2_^+^), and protonated imine (= NH^+^), respectively [33]. Compared to PANI-0 porous film, the quinonoid imine (=N–) peak disappears in the XPS N 1 s spectra from Cu-doped PANI films. This indicates that the copper ions doping induced the transformation of quinonoid imines in PANI films. The =N– structure in the Cu-doped PANI films is converted to a positively charged –N^**+**·^- structure, corresponding to the two peaks in the XPS spectra with binding energy greater than 400 eV (i.e., protonated amine and protonated imine) [34]. Specifically, with the increase in copper concentration from 0.005 M to 0.02 M, the percentage of the protonated amine and protonated imine increases from 50 to 66%, while the content of benzenoid amine decreases. It hints that the initial introduction of copper ions promotes protonation, leading to the formation of protonated amine. When the concentration is further increased to 0.03 M, PANI-3 porous film experiences a reverse trend, in which the content of protonated amine and protonated imine decreases to 52%.

As shown in Raman spectra, with the introduction of copper ions, the intensity of the peak at 1198 cm^−1^ increases, suggesting the increased oxidation degree of Cu-doped PANI porous films. Therefore, the decreased number of polarons for PANI-3 may be caused by the excessive oxidation degree of PANI-3, hindering the formation of carriers. Moreover, the dense microstructure of PANI-3 porous film was not conducive to carrier transport. These results indicate that the proper doping of copper ions for PANI may achieve the formation of Cu–N coordination, which facilitates the generation of polarons and bipolarons. However, exceeding the content of copper ions could format superfluous crystals complexes to hinder the delocalization of polarons and bipolarons. As mentioned above, the formation and the elimination of polarons and bipolarons delocalized on the PANI chains are the direct and most critical factors in realizing excellent emissivity modulation performance. Thus, based on the maximum number of polarons and bipolarons, the PANI-2 porous film is expected to exhibit the best infrared electrochromic performance.

**Fig. 7:**
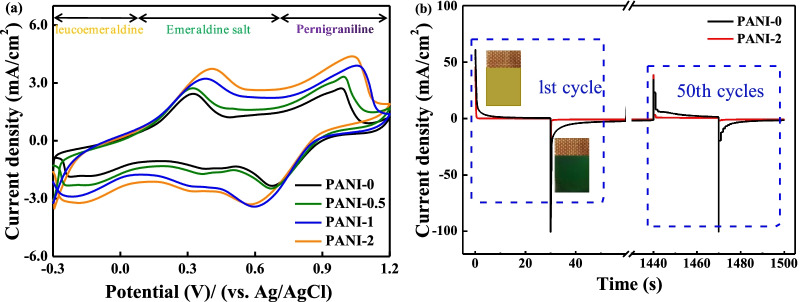
**a** The CV curves of Cu-doped PANI porous films measured in 0.5 M H_2_SO_4_ aqueous solutions with a scan rate of 50 mV/s sweeping between − 0.3 and 1.2 V. **b** The CA curves of 1st and 50th cycles and digital camera images of the PANI-0 and PANI-2 porous films at 0.5 V and − 0.25 V

According to the above structural analysis, it is anticipated the obvious differences in infrared emission modulation on the Cu-doped PANI films prepared with different copper ions concentrations. Cyclic voltammetric measurements were performed to preliminarily determine the electrochemical performance of the Cu-doped PANI porous films. As displayed in Fig. 7, the CV curves of Cu-doped PANI porous film present a pair of redox peaks corresponding to the state of emeraldine salt (ES) and leucoemeraldine (LE). The electrochemical active area under the CV curves is increasing with the increase in copper ions concentration. The PANI-2 porous film shows the largest electrochemical active area, suggesting the maximum amount of charge transfer and the potential optimal infrared electrochromic regulation performance.

As reported, the IR electrochromic properties of PANI-based films are directly related to their existential states [35, 36]. Hence, the potentials of − 0.25 V and 0.5 V were chosen on account of the films’ states completely transforming between LE and ES. Chronoamperometric (CA) experiment was conducted to investigate the IR electrochromic performance of PANI-2 and PANI-0 porous films. As shown in Fig. 7b, the color of the film materials changed from yellow to atrovirens in the voltage positive conversion process. For the PANI-2 porous film, the response time, defined as the times required for achieving 95% change of the full current density, from coloring state to bleaching state (the bias voltage − 0.25 V) can be calculated to be 0.7 s. When the bias voltage of 0.5 V is applied, the response time is determined to be 1.5 s (Additional file 1: Fig. S5(a)), much superior to that of pure PANI and previous reports [13, 14, 20, 37]. This can be reasonable by the reticular structure that provides more ion channels and a larger reactive area, improving the electrochemical reaction rate. The faster response time of the PANI-2 porous film suggests a more sensitive device. Furthermore, the response time of the present PANI-2 porous film is, respectively, about 1.3 s and 2.7 s for the bleaching state and the coloring state after 50 cycles of durability test, as shown in Fig. 7b and Additional file 1: Fig. S5(b). Therefore, the prepared PANI-2 films not only show excellent response ability, but also good stability.

**Fig. 8:**
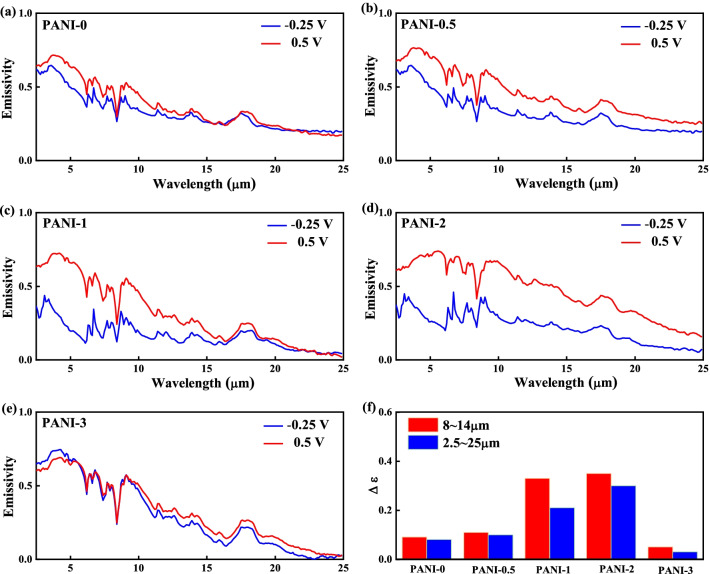
Emittance curves for **a** PANI-0, **b** PANI-0.5, **c** PANI-1, **d** PANI-2, and **e** PANI-3 porous films. **f** The ∆*ε* regions of the spectrum (2.5–25 µm and 8–12 µm) for Cu-doped PANI porous films at different Cu ions concentrations

To quantitatively analyze and compare the IR emissivity change of PANI-based films, the Fourier transform infrared spectra were studied in the whole wavelength range from 2.5 to 25 µm and the values of *ε* are calculated according to Eqs. (1) and (2). Figure 8 presents the emittance curves of Cu-doped PANI porous films at potentials of − 0.25 V and 0.5 V. The emittance curves at 0.5 V gradually rise with the copper ions concentration changing from 0.005 to 0.02 M, while a decrease is observed for the further increasing to 0.03 M. This trend is inconsistent with the number of polarons and bipolarons obtained in XPS results. As discussed above, the copper ions can activate the part of the inactive PANI film. Therefore, the Cu-doped PANI films, especially for PANI-1 and PANI-2 films, have low IR emissivity at -0.25 V. Similar to the emittance curves, the ∆*ε* between the curves at − 0.25 to 0.5 V presents a change in volcanic patterns. The maximum ∆*ε* from the PANI-2 porous film is 0.35 in ranges of wavelength 8–12 µm. By contrast, the PANI-0, PANI-0.5, PANI-1, and PANI-3 only provide the ∆*ε* of 0.1, 0.12, 0.21, and 0.06, respectively, as shown in Fig. 8e. The decreased ∆*ε* of PANI-3 may be caused by the dense microstructure of PANI-3 porous film, hindering the transmission of carriers [35, 38]. Therefore, the PANI-2 porous film exhibits the best-infrared modulation performance in this work (∆*ε* in the wavelength ranges of 2.5 to 25 µm). It indicates that the evolution of the emittance curves largely depends on the copper ions concentration and microstructures of PANI on the porous films.

**Fig. 9:**
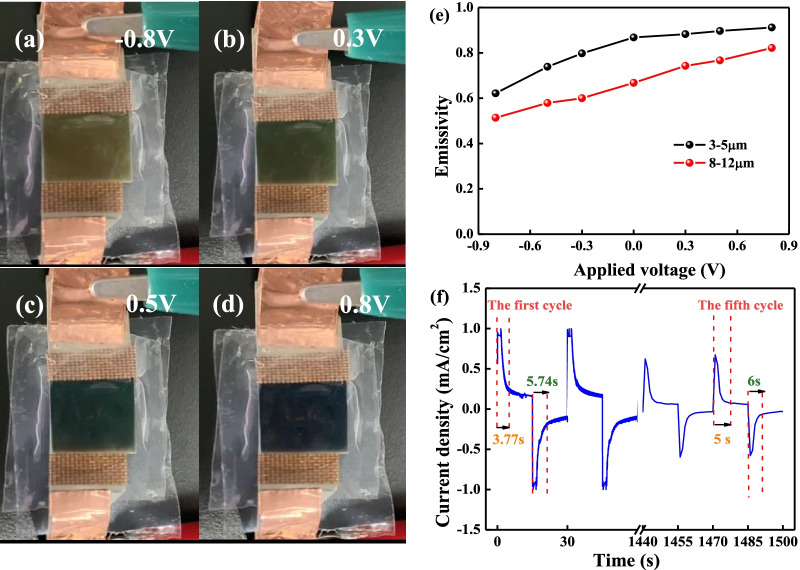
**a**–**d** Digital camera images of Cu-doped PANI IR electrochromic device at different voltages. **e** The emissivity value (*ε*) of Cu-doped PANI IR electrochromic device at regions of the spectrum (3–5 µm and 8–12 µm). **f** The CA curves of 1st and 50th cycles of the Cu-doped PANI IR electrochromic device at 0.8 V and -0.8 V

Based on the best Δ*ε* value, the PANI-2 porous film is selected as the functional layer to assemble IR electrochromic (EC) device. Figure 9a–d exhibits the digital photographs of the Cu-doped PANI IR EC device at different voltages. When the applied voltage is fixed at 0.3 V, the device presents a color change from yellow to green. As the applied voltage increases to 0.5 V, the color of the device changes to dark green. When the applied voltage reaches 0.8 V, the device presents atrovirens. The results demonstrate that the Cu-doped PANI IR devices can tunably blend with green or yellow backgrounds under different voltages.

To explore the application potential of the Cu-doped PANI IR EC device in optical and thermal management, the IR emissivity of the EC device was also investigated in the wavelength ranges of 3–5 µm and 8–12 µm at the different voltages. As shown in Fig. 9e, the evolution of *ε* in two wavelength ranges shows a similar tendency. That is, the emissivity gradually increases along with the applied voltage. When the applied voltage reaches 0.5 V, the emissivity of the device increases a little and tends to be stable as a result of the PANI layer in the ES state. Note that the present △*ε* of 0.32 in 8–12 µm is much larger than that (0.24) from the previous pure sulfuric acid-doped PANI device [15]. In addition, the response time and stability of the device are also shown in Fig. 9f. The coloring time of the device is about 5.74 s (1st cycle) and 6 s (50th cycle), while the fading time is about 3.77 s (1st cycle) and 5 s (50th cycle). Meanwhile, the current density of the device still retains less than 0.3 mA cm^−2^ loss after the 50 cycles. Therefore, the present IR electrochromic device exhibits superior tenability and good electrochromic stability.

## Conclusions

In summary, various Cu-doped PANI porous films have been prepared via the electropolymerization procession of the surfaces of Au/porous substrates. Based on the electronic structure and appropriate porous structure, the Cu-doped PANI porous film prepared at the concentration of 0.02 M showed the optimal IR electrochromic properties and the best emissivity modulation. Furthermore, a flexible IR electrochromic device has been fabricated and shows good modulation of the emittance variation in the wavelength ranges of 8–12 µm. The results in this work will not only provide new insights into the redox states and protonation levels of the PANI, but also a new thought for enhancing the IR emissivity modulation ability of electrochromic films. This will promote the applications of IR electrochromic devices in military camouflage and thermal control of satellites.

## Supplementary Information

**Additional file 1. Fig. S1.** The polymerization current as a function of time under different copper content for PANI porous films. **Fig. S2.** SEM images of surface on (**a**) PES, (**b**) Au/PES membrane, (**c**) PANI-0 porous films, and (**d**) cross sections of the PANI-0 porous films. **Fig. S3.** AFM images of surface on (**a**) PANI-0 porous films (**b**) PANI-0.5, (**c**) PANI-1, (**d**) PANI-2, and (**e**) PANI-3 porous films. **Fig. S4.** XPS spectra of: (**a**) survey spectrum, and XPS spectra of S 2p spectrum for (**b**) PANI-0, (**c**) PANI-0.5, (**d**) PANI-1, (**e**) PANI-2, and (**f**) PANI-3 porous films. **Fig. S5.** Response time of PANI-0 and PANI-2 porous films at 0.5 V and -0.25 V (**a**) 1st cycle, and (**b**) 50th cycles. **Fig. S6.** (**a**) Nyquist plots by EIS technique for prepared porous films. (**b**) The charge transfer resistance (Rct) and the series resistance (Rs) of prepared porous films

## Data Availability

All the data and material are available in the manuscript.

## Abbreviations

IR: Infrared
PANI: Polyaniline
*ε*: Emissivity
∆*ε*: Emittance variation
EC: Electrochromic
CPs: Conducting polymers
CSA: Camphor sulfonic acid
PES: Polyethersulfone
PE: Polyethylene
PVA: Polyvinyl alcohol
SEM: Scanning electron microscopy
AFM: Atomic force microscope
XRD: X-ray diffraction
XPS: X-ray photoelectron spectroscopy
CV: Cyclic voltammetric
CA: Chronoamperometric
*h*: Plank constant
*c*: Speed of light
*λ*: Wavelength
*T*: Temperature
*B*(*λ*): Spectral radiance of the black body at λ
*R*(*λ*): Reflectivity at λ
H: Hydrogen
N: Nitrogen
*i*–*t* curves: Chronoamperometry method curves
2D: Two-dimensional
3D: Three-dimensional
EDS: Energy-dispersive spectrometer
ES: Emeraldine salt
LE: Leucoemeraldine
