# Supplementary material for: Enhancement of Electrochromic Properties of Polyaniline Induced by Copper Ions
Source: Nanoscale Res Lett. 2022 May 12;17:51. doi: 10.1186/s11671-022-03689-1 (PMC9098743; doi:10.1186/s11671-022-03689-1)
Supplement: Supplementary file 1 — Additional file 1. Fig. S1. The polymerization current as a function of time under different copper content for PANI porous films. Fig. S2. SEM images of surface on (a) PES, (b) Au/PES membrane, (c) PANI-0 porous films, and (d) cross sections of the PANI-0 porous films. Fig. S3. AFM images of surface on (a) PANI-0 porous films (b) PANI-0.5, (c) PANI-1, (d) PANI-2, and (e) PANI-3 porous films. Fig. S4. XPS spectra of: (a) survey spectrum, and XPS spectra of S 2p spectrum for (b) PANI-0, (c) PANI-0.5, (d) PANI-1, (e) PANI-2, and (f) PANI-3 porous films. Fig. S5. Response time of PANI-0 and PANI-2 porous films at 0.5 V and -0.25 V (a) 1st cycle, and (b) 50th cycles. Fig. S6. (a) Nyquist plots by EIS technique for prepared porous films. (b) The charge transfer resistance (Rct) and the series resistance (Rs) of prepared porous films [file 11671_2022_3689_MOESM1_ESM.docx]

**Supporting Information**

Enhancement of electrochromic properties of polyaniline induced by copper ions

Ting Qin^1^, Lianwen Deng^1^, Pin Zhang^2^*, Min Tang^1^, Chen Li^1^, Haipeng Xie^1^, Shengxiang Huang^1^, Xiaohui Gao^1^*

*1 School of Physics and Electronics, Central South University, Changsha, 410083, China*

*2 National Key Laboratory on Electromagnetic Environmental Effects and Electro-optical Engineering, Army Engineering University of PLA, Nanjing 210007, China*

* Corresponding author. E-mail: [182211037@csu.edu.cn](mailto:182211037@csu.edu.cn); [xiaohuigao@csu.edu.cn](mailto:xiaohuigao@csu.edu.cn;)

Fig. S1 The polymerization current as a function of time under different copper content for PANI porous films.

Fig. S2 SEM images of surface on (a) PES, (b) Au/PES membrane, (c) PANI-0 porous films, and (d) cross sections of the PANI-0 porous films.

Fig. S3 AFM images of surface on (a) PANI-0 porous films (b) PANI-0.5, (c) PANI-1, (d) PANI-2, and (e) PANI-3 porous films.

Fig. S4 XPS spectra of: (a) survey spectrum, and XPS spectra of S 2p spectrum for (b) PANI-0, (c) PANI-0.5, (d) PANI-1, (e) PANI-2, and (f) PANI-3 porous films.

Fig. S5 Response time of PANI-0 and PANI-2 porous films at 0.5 V and -0.25 V (a) 1st cycle, and (b) 50th cycles.

Fig. S6 (a) Nyquist plots by EIS technique for prepared porous films. (b) The charge transfer resistance (*R_ct_*) and the series resistance (*R_s_*) of prepared porous films.
